# Microarray Expression Data Identify *DCC* as a Candidate Gene for Early Meningioma Progression

**DOI:** 10.1371/journal.pone.0153681

**Published:** 2016-04-20

**Authors:** Hans-Juergen Schulten, Deema Hussein, Fatima Al-Adwani, Sajjad Karim, Jaudah Al-Maghrabi, Mona Al-Sharif, Awatif Jamal, Fahad Al-Ghamdi, Saleh S. Baeesa, Mohammed Bangash, Adeel Chaudhary, Mohammed Al-Qahtani

**Affiliations:** 1 Center of Excellence in Genomic Medicine Research, King Abdulaziz University, Jeddah, Saudi Arabia; 2 KACST Technology Innovation Center in Personalized Medicine, King Abdulaziz University, Jeddah, Saudi Arabia; 3 King Fahad Medical Research Center, King Abdulaziz University, Jeddah, Saudi Arabia; 4 Department of Biology, King Abdulaziz University, Jeddah, Saudi Arabia; 5 Department of Pathology, Faculty of Medicine, King Abdulaziz University Hospital, Jeddah, Saudi Arabia; 6 Department of Pathology, King Faisal Specialist Hospital and Research Center, Jeddah, Saudi Arabia; 7 Division of Neurosurgery, Department of Surgery, King Abdulaziz University Hospital, Jeddah, Saudi Arabia; University of South Alabama Mitchell Cancer Institute, UNITED STATES

## Abstract

Meningiomas are the most common primary brain tumors bearing in a minority of cases an aggressive phenotype. Although meningiomas are stratified according to their histology and clinical behavior, the underlying molecular genetics predicting aggressiveness are not thoroughly understood. We performed whole transcript expression profiling in 10 grade I and four grade II meningiomas, three of which invaded the brain. Microarray expression analysis identified deleted in colorectal cancer (*DCC*) as a differentially expressed gene (DEG) enabling us to cluster meningiomas into *DCC* low expression (3 grade I and 3 grade II tumors), *DCC* medium expression (2 grade I and 1 grade II tumors), and *DCC* high expression (5 grade I tumors) groups. Comparison between the *DCC* low expression and *DCC* high expression groups resulted in 416 DEGs (*p*-value < 0.05; fold change > 2). The most significantly downregulated genes in the *DCC* low expression group comprised *DCC*, phosphodiesterase 1C (*PDE1C*), calmodulin-dependent 70kDa olfactomedin 2 (*OLFM2*), glutathione S-transferase mu 5 (*GSTM5*), phosphotyrosine interaction domain containing 1 (*PID1*), sema domain, transmembrane domain (TM) and cytoplasmic domain, (semaphorin) 6D (*SEMA6D*), and indolethylamine N-methyltransferase (*INMT*). The most significantly upregulated genes comprised chromosome 5 open reading frame 63 (*C5orf63*), homeodomain interacting protein kinase 2 (*HIPK2*), and basic helix-loop-helix family, member e40 (*BHLHE40*). Biofunctional analysis identified as predicted top upstream regulators beta-estradiol, TGFB1, Tgf beta complex, LY294002, and dexamethasone and as predicted top regulator effectors NFkB, PIK3R1, and CREBBP. The microarray expression data served also for a comparison between meningiomas from female and male patients and for a comparison between brain invasive and non-invasive meningiomas resulting in a number of significant DEGs and related biofunctions. In conclusion, based on its expression levels, *DCC* may constitute a valid biomarker to identify those benign meningiomas at risk for progression.

## Introduction

Meningiomas arise from arachnoidal cap cells of the arachnoidal membrane and are the most common intracranial neoplasms representing 20–35% of all primary brain tumors [1]. Meningiomas are histologically classified as benign (WHO grade I), atypical (WHO grade II), or anaplastic (WHO grade III) tumors comprising approximately 80%, 15%, and 5% of the cases, respectively. WHO grade I meningiomas are categorized into nine histological subtypes of which the meningothelial subtype is the most frequent one. Meningiomas are commonly cured by surgical treatment and for aggressive tumors additional treatment is advised. Besides existing histopathological criteria, predictive biomarkers for meningioma progression are not established yet. To improve prediction for the clinical behavior of meningiomas, a new scoring system for risk stratification had been proposed, based on age, WHO grade, cytogenetic profile, tumor size, and tumor location [2].

About half of the sporadic meningiomas harbor a mutation in the tumor suppressor gene NF2 encoding merlin that is a critical factor for regulation of contact-dependent inhibition of cell proliferation. The majority of grade I meningiomas exhibit a cytogenetically normal chromosome status whereas a minority is characterized by -22q and/or -1p [3]. In subsets of grade II meningiomas, additional chromosomal imbalances are common, including -6q, -14q, and -18q [3]. Chromosomal imbalances in grade III meningiomas can be more complex and may include -9p21 and -10. Besides the association of -22q with the impairment of NF2 function, impact of other chromosome arm losses are not thoroughly understood in the molecular etiology of meningiomas.

Deleted in Colorectal Cancer (*DCC*) is a member of the immunoglobulin superfamily and encodes a type I transmembrane receptor for netrin-1 and a number of other ligands including cerebellin precursor protein 4 (*CBLN4*) [4, 5]. The mature DCC protein contains an extracellular, a transmembrane, and a cytoplasmatic domain that contains a segment for mediating apoptotic signals. Crystal structure experiments indicated that DCC multimerizes via its extracellular FN type III domains 5 and 6 with netrin-1 [6]. Inhibition of netrin-1 induces multimerization of DCC or increases apoptosis mediated by a truncated DCC variant [7]. The netrin-1/DCC signaling complex has the capacity to activate a number of intracellular signal cascades that, depending on the cellular context, results for example in rearrangement of the cytoskeleton, vasculogenesis, or morphogenetic processes [8]. In the CNS, *DCC* is comparably highly expressed and a critical factor for axon guidance and neuronal migration. Recently, cell culture experiments demonstrated that netrin-1 and DCC are regulators of somatic cell reprogramming to pluripotency [9].

The well established tumor suppressor function of DCC is a result of its capacity to induce apoptosis as a dependence receptor for netrin-1 in case a ligand-receptor binding is interfered or the ligand is not present. This was demonstrated *in vivo* in netrin-1 knockout mice revealing that regulation of apoptosis is depending on the availability of netrin-1 for DCC [10, 11]. Furthermore, loss of DCC in mice bearing an inactivated TP53 gene promoted metastatic capacity [12] whereas induced DCC expression in glioblastoma cell lines inhibited spontaneous cell migration [13]. Consistent with its tumor suppressor function, downregulation of DCC expression has been demonstrated in a number of cancer types including colorectal [14], ovarian, pancreatic, hepatocellular carcinomas [15], and neuroblastoma [16]. In different histological types of gliomas, a number of mechanisms has been described that alter DCC expression on the transcriptional or protein level that in case of transcriptional downregulation or negative immunoreactivity is related to high grade and progressive tumors [17–19]. In our study, we employed whole transcript microarrays to identify molecular biomarkers for early meningioma progression. Limited numbers of samples, as in our study, have been successfully used in other microarray expression studies on meningiomas to detect tumor-related gene profiles [20, 21].

## Material and Methods

### Tumor samples

The meningioma specimens studied were derived from patients who were treated surgically between April 2013 and January 2015 at the King Abdulaziz University Hospital, Jeddah. Written informed consent from the donors or the next of kin was obtained for the use of samples in research. The study was approved by the Research Ethics Committee of the King Abdulaziz University, Faculty of Medicine, #976–12. Histopathological diagnosis was performed by a team of pathologists (JM, AJ, and FG).

### RNA and array processing

Isolation of total RNA and array sample processing were performed from in RNAlater (Qiagen, Hilden, Germany) preserved tumor specimens as described earlier [22]. In brief, the Agilent 2100 Bioanalyzer (Agilent Technologies, Palo Alto, CA) (11 cases) was employed to assess RNA integrity. The assigned RNA integrity number was for all samples > 5. The NanoDrop ND-1000 spectrophotometer (NanoDrop Technologies, Wilmington, DE) was utilized to determine RNA concentration. All RNA samples were processed using the Ambion WT Expression Kit (Life Technologies, Austin, TX), the GeneChip WT Terminal Labeling and Controls Kit (Affymetrix, Santa Clara, CA), and the Affymetrix GeneChip Hybridization, Wash and Stain Kit. The processed samples were hybridized to whole transcript Affymetrix Human Gene 1.0 ST GeneChip arrays that interrogate with a set of 764,885 probes 36,079 annotated reference sequences (NCBI build 36). The microarrays were scanned on a GeneChip Scanner 3000 7G and probe cell intensity data (CEL) files were generated using the GeneChip Command Console (AGCC) software. Microarray data have been deposited at the NCBI’s Gene Expression Omnibus under accession number GSE77259.

### Gene and exon expression analysis

For gene and exon expression analysis, CEL files were imported into Partek Genomics Suite version 6.6 (Partek Inc., MO) using the default Robust Multi-array Average (RMA) settings for normalization. CEL files from three brain normal (BN) samples were imported from the Affymetrix Data Resource Center (http://www.affymetrix.com/support/mas/datasets.affx) and served as gene expression reference. Lists of differentially expressed genes (DEGs) were generated by applying analysis of variance (ANOVA) and using a *p*-value ˂ 0.05 and a fold change > 2. Where indicated, a *p*-value with a false discovery rate (FDR) (Step up method) < 0.05 and a fold change > 2.0 were employed. Quality of experiments was assessed on the basis of the QC metrics table and QC graphical report. Overall variance in gene expression between samples or groups of samples was assessed by principal component analysis (PCA). Average linkage hierarchical clustering was carried out using Spearman’s correlation as a similarity matrix. The Partek Gene Ontology (GO) enrichment tool was employed to group DEGs into functional categories that were ranked according to their *p*-values.

### Functional network and pathway analysis

The Ingenuity Pathways Analysis software (IPA; build version 338830M) (Ingenuity Systems, Redwood City, CA) was employed to interpret biological significance of expression data and using the Ingenuity Knowledge Base as reference data set. Direct and indirect molecular relationships were included in analysis settings. Fisher`s exact test *p*-values indicate significance of relationships between the analyzed data set genes/molecules (both items were used interchangeably in the context of IPA) and the functional frameworks prebuilt or generated *de novo* by IPA. The Molecule Activity Predictor was utilized, as specified in the prediction legends of figures, to predict expression effects/coherence of expression effects of a molecule on other network molecules. Upstream regulators analysis was employed to explain how differences in target gene expression are regulated by upstream molecules and what kind of biological activities are involved. The overlap *p-*value is a statistical measure to indicate extent of overlap between the uploaded data set and genes that are known to be regulated by an upstream regulator. The regulator effects network analysis was employed to explain which regulators target DEGs from the uploaded data set and what kind of downstream effects, i.e. diseases and/or functions are connected. In how far a generated network is consistent with the Knowledge Base is scaled by a consistency score.

### Semiquantitative RT-PCR

Expression of *DCC* was assessed by semiquantitative RT-PCR using housekeeping gene *B2M* as reference. Primer sequences used were *DCC* exon 28 forward, 5`TGAAGTGTCTGAGGAGAG-3`, *DCC* exon 29 reverse, 5`- GGTATGCTGCAAAGTTCC-3`, *B2M* forward, 5`- TCATCCAGCAGAGAATGG-3`, and *B2M* reverse, 5`- GAGATAGAAAGACCAGTCCT-3`. RT-PCR analysis was performed in 18 μL volumes containing each 2 μL buffer mix, 0.1% 2-mercaptoethanol, 0.0125% BSA, 3 mM MgCl_2_, 10 nmol of each dNTP, 5 pmol forward primers, 5 pmol reverse primers, 2.5 units GoTaq DNA Polymerase (Promega, Madison, WI), and 250 ng of second strand cDNA template that was generated using the Ambion WT Expression Kit. No cDNA template was included in negative control. The standard PCR protocol comprised an initial denaturation step at 95°C followed by 5 touch-down cycles with an annealing temperature decreasing 1°C per cycle from 65°C to 61°C. Subsequently, 24 cycles followed with 30 sec at 95°C, 30 sec at 60°C, and 30 sec at 72°C. The final step was performed for 10 min at 72°C. PCR products were resolved by electrophoresis on a 3% agarose gel. Product size for *DCC* was 187 bp and for *B2M* 156 bp. Gel band densities were measured using ImageJ 1.49v [23].

## Results

The 14 analyzed meningiomas comprised 10 WHO grade I tumors and four WHO grade II tumors, one of which was a recurrence (Table 1). Four grade I tumors were classified according to their subtypes as meningothelial, three as transitional, two as fibroblastic, and one as psammomatous. Three of the four grade II tumors were brain invasive. Mean age of the study group was 54.1±10.1 years. Four patients were males and 10 were females.

**Table 1:** Demographic, histopathological, and *DCC* expression characteristics of 14 meningiomas.

Case	Age (year)	Gender^1^	Histology	Location	WHO^2^ grade	*DCC* expression	Fold change^3^
Jed13_MN	50	F	meningothelial	left frontal	I	high	2.16
Jed26_MN	59	M	meningothelial	occipital	I	high	1.61
Jed40_MN	64	F	transitional	cerebellopontine angle	I	high	1.37
Jed43_MN	55	F	psammomatous	spinal	I	high	1.83
Jed70_MN	73	F	fibroblastic	right temporal	I	high	1.18
Jed04_MN	57	F	transitional	left tentorial	I	medium	-1.88
Jed53_MN	64	F	transitional with brain invasion	right sphenoid wing	II	medium	-1.71
Jed57_MN	48	F	transitional	right parasagittal	I	medium	-1.65
Jed18_MN	64	F	meningothelial with brain invasion	left temporal	II	low	-5.59
Jed34_MN	41	M	meningothelial	left sphenoid wing	I	low	-5.00
Jed36_MN	36	M	meningothelial	right frontoparietal	I	low	-7.99
Jed64_MN	49	F	fibroblastic	right frontal	I	low	-7.51
MN_Jed49	51	F	atypical with brain invasion	posterior right subfalcine (recurrence)	II	low	-6.40
Jed58_MN	47	M	atypical	right falcine	II	low	-5.57

^1^ M, male; F, female;

^2^ WHO, World Health Organization;

^3^ fold change of *DCC* expression in relation to expression of the three normal brain samples.

### Microarray expression analysis of *DCC*

**Fig 1:**
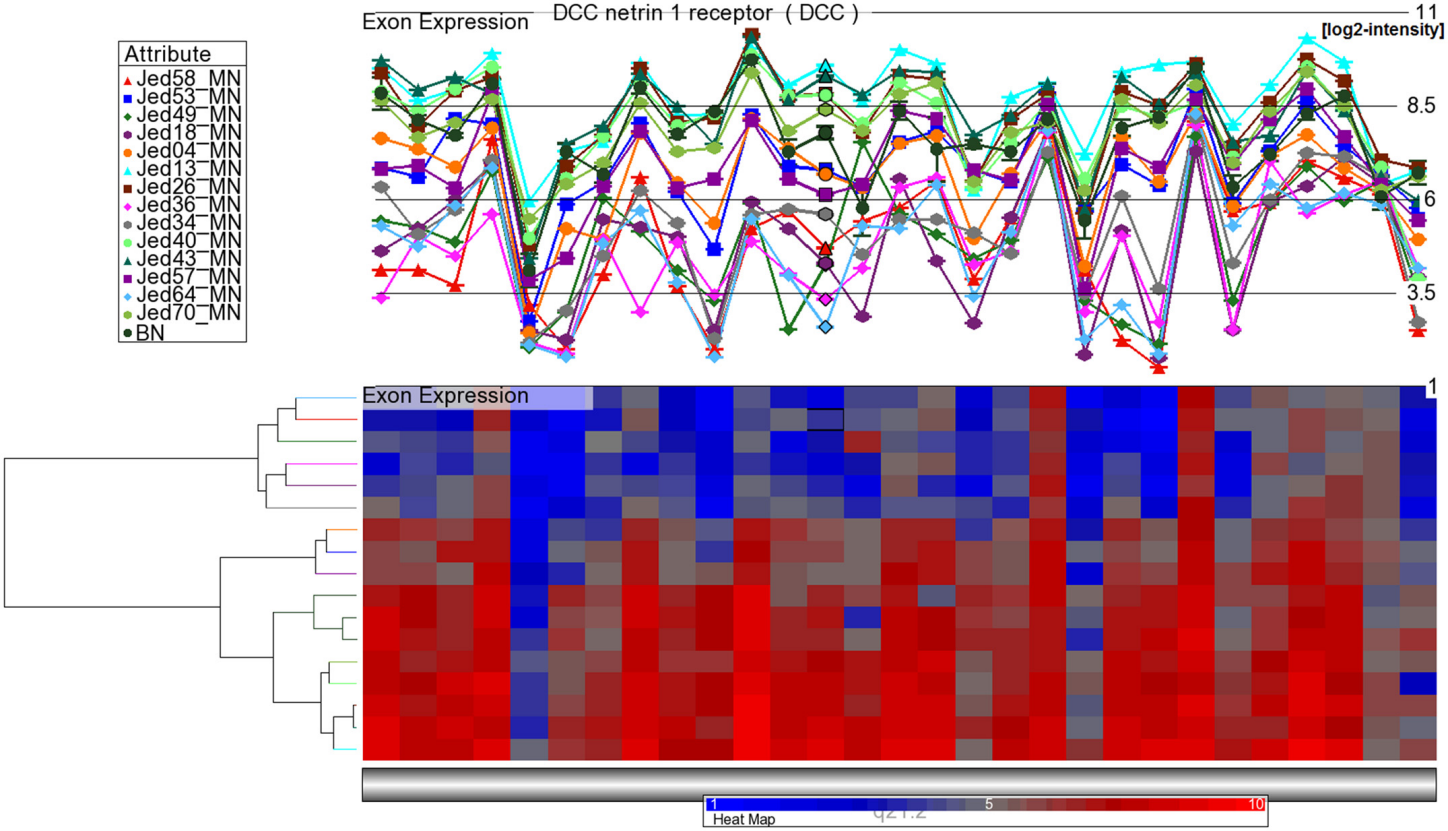
Expression of *DCC* exons in 14 meningiomas and three normal brain samples. **Upper panel, DCC exons are interrogated with 29 probes.** Lower panel, hierarchical cluster analysis based on the *DCC* expression values reveals two main branches, one of which contains the six *DCC* low expression meningiomas. Based on the expression values (Table 1) the meningiomas were grouped into *DCC* high, *DCC* medium, and *DCC* low expression samples.

**Fig 2:**
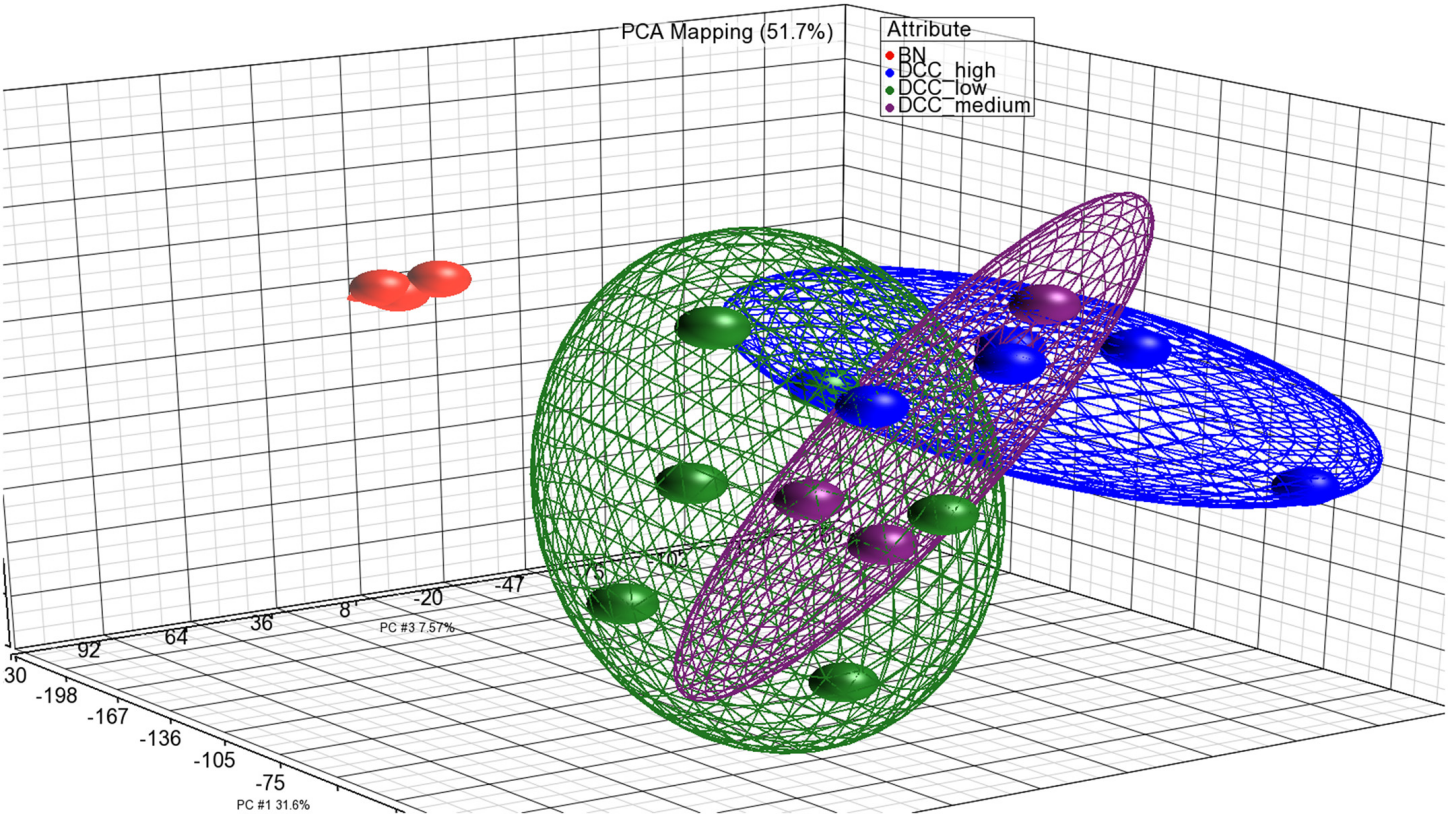
PCA scatter plot as a dimensional measure for the similarity of the expression profiles of samples (colored dots). Ellipsoids represent the 95% confidence interval and are a measure for the distance of relationships between samples of a group. Green, *DCC* low expression; purple, *DCC* medium expression; blue, *DCC* low expression; red, normal brain samples (BN).

**Fig 3:**
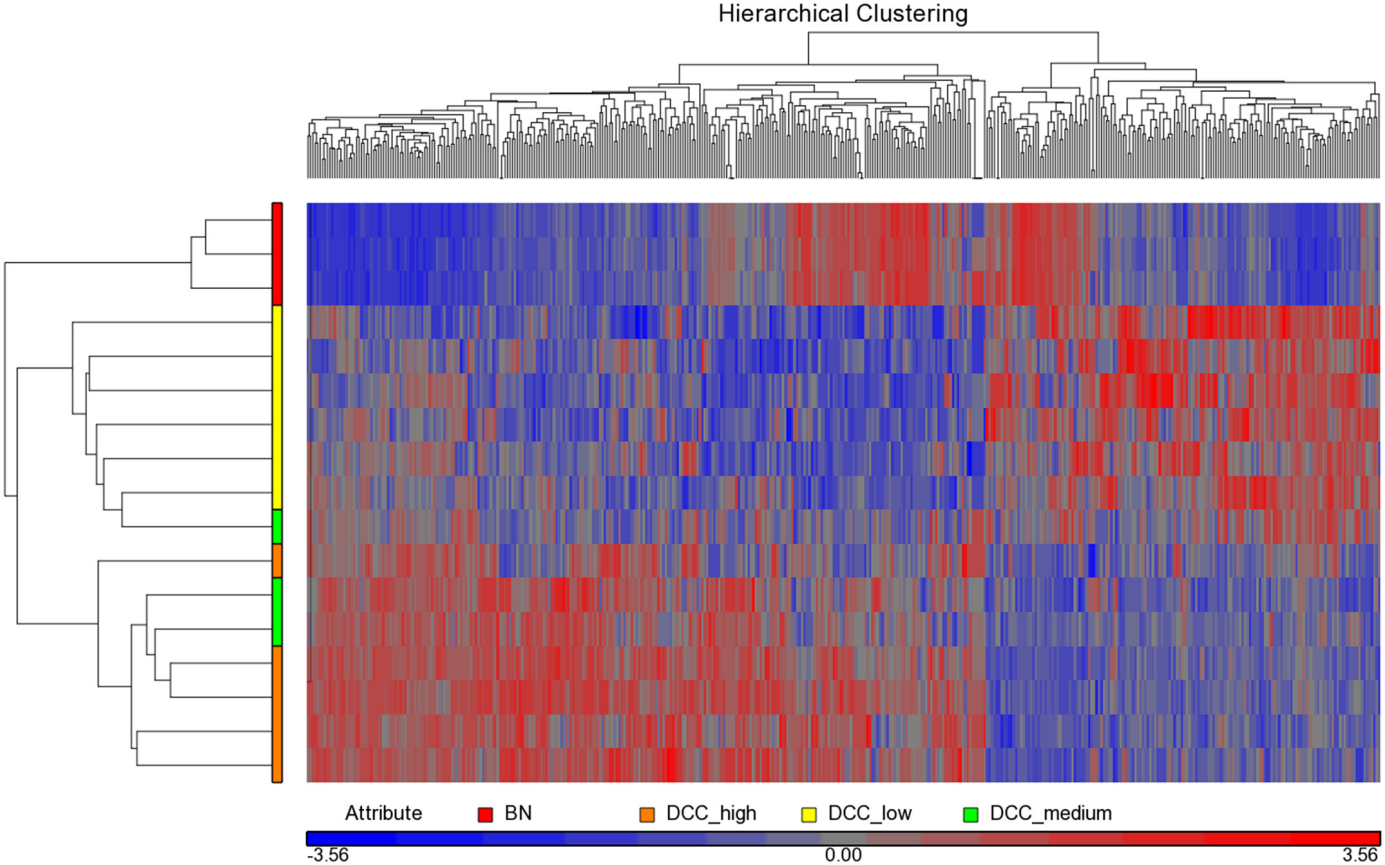
Unsupervised hierarchical cluster analysis of 416 genes that were differentially expressed (*p*-value < 0.05; fold change > 2.0) between the three *DCC* expression groups. BN samples are included in cluster analysis. A number of genes is represented by more than one transcript. Meningiomas are clustering into two main branches, one of which contains the *DCC* low expression samples and a *DCC* medium expression sample that was a brain invasive case. Color scheme bar indicates comparably higher and lower expression values in red and blue color, respectively. Color scheme for samples: yellow, *DCC* low expression; green, *DCC* medium expression; orange, *DCC* high expression; BN samples, red.

We noticed *DCC* as a DEG when we applied ANOVA to compare each of the 14 meningiomas with the group of three BN samples to assess variation in expression levels among the tumors and between tumors and BN samples. We analyzed *DCC* in more detail based on its known critical function as a netrin-1 dependence receptor and its implications in various cellular key functions including axon guidance, cell migration, and pro-apoptotic activities; the latter known to be impaired in numerous cancer types. An exon expression plot and a corresponding heat map revealed that *DCC* exon expression levels varied widely between the samples (Fig 1). The resulting gene expression levels enabled us to categorize the meningiomas either as *DCC* low, *DCC* medium, or *DCC* high expression tumors (Table 1). A PCA scatter plot visualizes the relationship of expression of the different *DCC* groups and BN samples in a 3 dimensional space (Fig 2). ANOVA carried out between the *DCC* low expression and *DCC* high expression groups generated 416 DEGs. Unsupervised hierarchical cluster analysis on the 416 DEGs categorized the three *DCC* groups and BN samples into different branches (Fig 3). Detailed analysis of the 416 DEGs revealed 260 downregulated and 156 upregulated genes in the *DCC* low expression compared to the *DCC* high expression group (S1 Table). The most significantly downregulated genes in the *DDC* low expression group comprised, besides *DCC*, phosphodiesterase 1C (*PDE1C*), calmodulin-dependent 70kDa olfactomedin 2 (*OLFM2*), glutathione S-transferase mu 5 (*GSTM5*), phosphotyrosine interaction domain containing 1 (*PID1*), sema domain, transmembrane domain (TM) and cytoplasmic domain, (semaphorin) 6D (*SEMA6D*), and indolethylamine N-methyltransferase (*INMT*). The most significantly upregulated genes comprised chromosome 5 open reading frame 63 (*C5orf63*), homeodomain interacting protein kinase 2 (*HIPK2*), and basic helix-loop-helix family, member e40 (*BHLHE40*). Significance for these deregulated genes was based on a *p*-value with FDR < 0.05 and a fold change > 2. An RT-PCR analysis on the five *DCC* high expression and six *DCC* low expression meningiomas substantially supported the microarray expression data (S1 Fig).

### Biofunctional analysis of *DCC* low *vs*. *DCC* high expression

**Fig 4:**
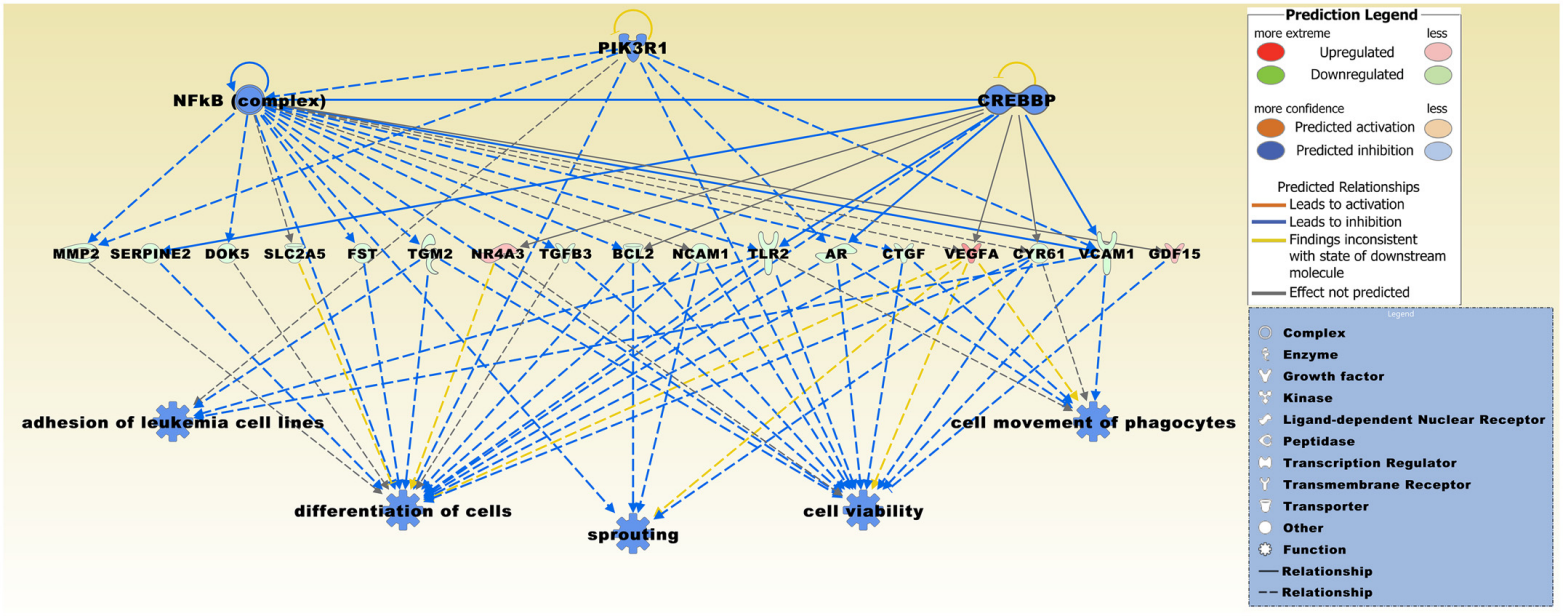
The predicted top regulator effects network with a consistency score of 8.489 in the *DCC* low *vs*. *DCC* high expression comparison. Upstream regulators NFkB, PIK3R1, and CREBBP target a number of DEGs including *MMP2*, *SERPINE2*, *DOK5*, *SLC2A5*, *FST*, *TGM2*, *NR4A3*, *TGFB3*, *BCL2*, *NCAM1*, *TLR2*, *AR*, *CTGF*, *VEGFA*, *CYR61*, *VCAM1*, and *GDF15*. Connected downstream functions are entitled adhesion of leukemia cell lines, differentiation of cells, sprouting (including cell morphological characteristics), cell viability, and cell movement of phagocytes.

A GO enrichment analysis performed on the 416 DEGs displayed significantly overrepresented functional groups in the three categories cellular component, molecular functions, and biological process (S2 Fig). The two mostly enriched functions in the categories were extracellular matrix and extracellular region (cellular component), chemoattractant activity and molecular transducer activity (molecular functions), and biological adhesion and developmental process (biological process). The predicted top five upstream regulators for the 416 DEGs were beta-estradiol, TGFB1, TGF beta family, LY294002 which is an inhibitor of PI3Ks, and the anti-inflammatory and immunosuppressive steroid dexamethasone (S3 Fig). In the predicted top regulator effects network the upstream effectors were NFkB (complex), PIK3R1, and CREBBP that target a number of DEGs, i.e. *MMP2*, *SERPINE2*, *DOK5*, *SLC2A5*, *FST*, *TGM2*, *NR4A3*, *TGFB3*, *BCL2*, *NCAM1*, *TLR2*, *AR*, *CTGF*, *VEGFA*, *CYR61*, *VCAM1*, and *GDF15* (Fig 4). Downstream network functions included adhesion of leukemia cell lines, differentiation of cells, sprouting, cell viability, and cell movement of phagocytes.

### Meningiomas from females *vs*. from males

**Fig 5:**
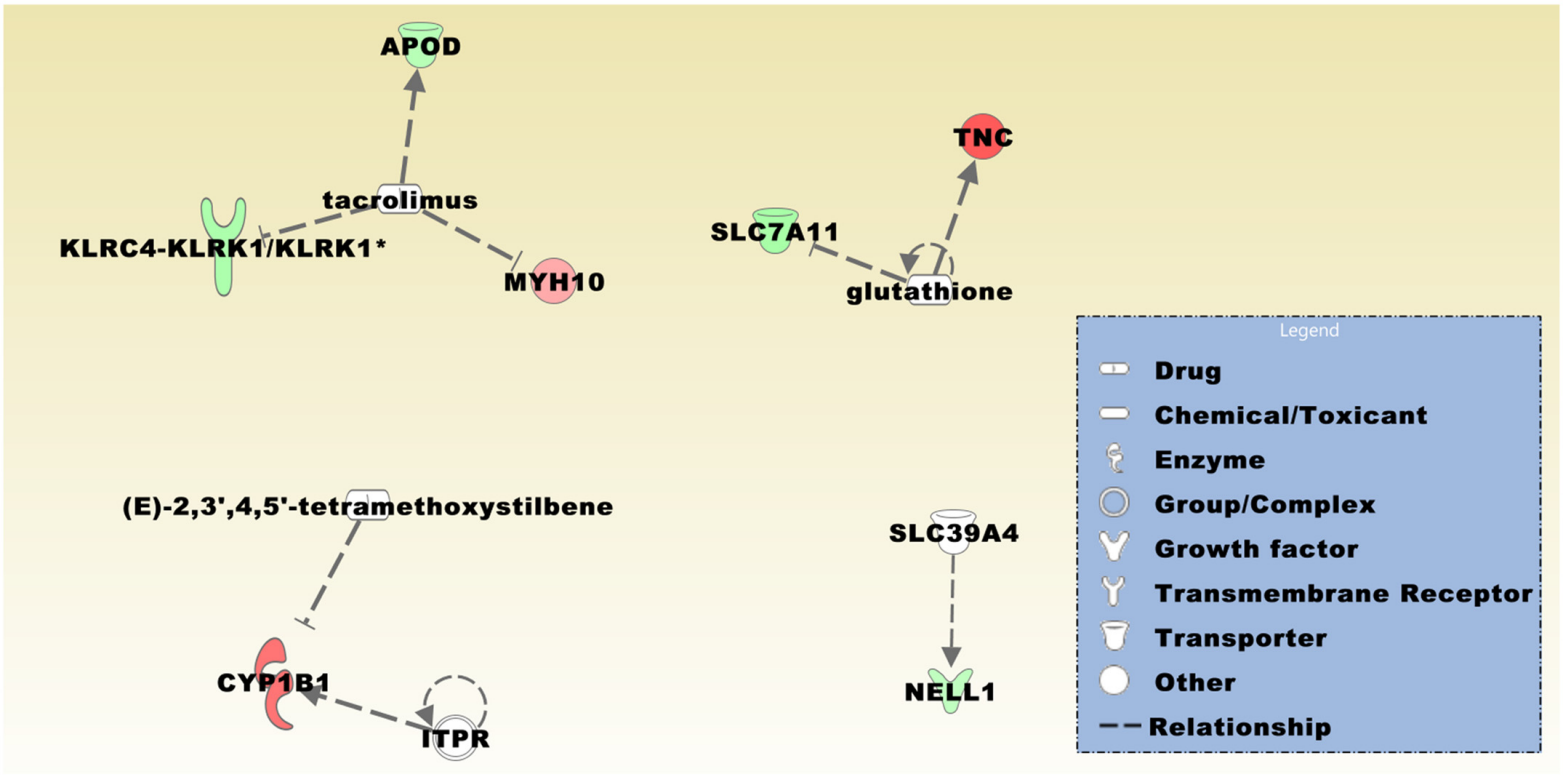
The predicted top upstream regulators in the comparison group female *vs*. male meningioma patients are tacrolimus, glutathione, ITPR, (E)-2,3',4,5'-tetramethoxystilbene, and SLC39A4 with a *p*-value of overlap of 1.16E-03, 1.55E-03, 2.02E-03, 2.02E-03, and 2.02E-03, respectively. Target genes are *APOD*, *KLRC4-KLRK1*/*KLRK1*, *MYH10*, *TNC*, *SLC7A11*, *CYP1B1*, and *NELL1*. Upregulated and downregulated genes in red and blue color, respectively. Asterisk indicates a gene that is represented in the dataset by more than one transcript.

To identify genes that are related to the overrepresentation of meningiomas in females, we compared the expression profiles of the 10 female with the four male cases resulting in a list of 43 DEGs (S2 Table). Among genes that were higher expressed in meningiomas from females were rhotekin 2 (*RTKN2*), neuritin 1 (*NRN1*), small nucleolar RNA, C/D box 114–26 (*SNORD114-26*), and leucine rich adaptor protein 1-like (*LURAP1L*). The gene list also contains 11 genes that are expressed from the Y chromosome. Autosomal genes that were comparably lower expressed in meningiomas from females included EPH receptor A3 (*EPHA3*), parvalbumin (*PVALB*), calbindin 2 (*CALB2*), and solute carrier family 38 member 3 (*SLC38A3*). Top upstream regulators comprised the calcineurin inhibitor tacrolimus, glutathione, ITPR, the resveratrol analogue (E)-2,3',4,5'-tetramethoxystilbene, and the zinc transporter SLC39A4 (Fig 5).

### Brain invasive *vs*. non-invasive meningiomas

**Fig 6:**
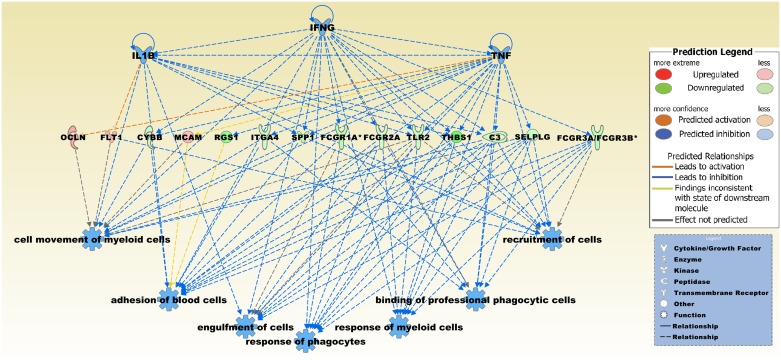
The predicted top regulator effects network with a consistency score of 13.0 in the brain invasive *vs*. non-invasive meningioma dataset. Effector molecules IFNG, IL1B, and TNF target a number of DEGs including *OCLN*, *FLT1*, *CYBB*, *MCAM*, *RGS1*, *ITGA4*, *SPP1*, *FCGR1A*, *FCGR2A*, *TLR2*, *THBS1*, *C3*, *SELPLG*, and *FCGR3A*/*FCGR3B*. Connected downstream functions are entitled, cell movement of myeloid cells, adhesion of blood cells, engulfment of cells, response of phagocytes, response of myeloid cells, binding of professional phagocytic cells, and recruitment of cells. Upregulated and downregulated genes in red and blue color, respectively. Asterisk indicates a gene that is represented in the dataset by more than one transcript.

To identify an expression pattern that is related to meningioma invasion, we compared the three invasive meningiomas with the remaining 11 non-invasive meningiomas (Table 1). This comparison resulted in a set of 256 DEGs of which 94 were comparably down- and 162 upregulated in the invasive tumors (S3 Table). Among the most significantly upregulated genes in the invasive group were coiled-coil domain containing cell adhesion molecule L1-like (*CHL1*), RNA binding protein, fox-1 homolog (C. elegans) 3 (*RBFOX3*), peroxisomal biogenesis factor 5-like (*PEX5L*), and RAB3D, member RAS oncogene family (*RAB3D*), The most significantly downregulated genes comprised solute carrier family 2 (facilitated glucose transporter), member (*SLC2A12*), ABI family, member 3 (NESH) binding protein (*ABI3BP*), selectin P ligand (*SELPLG*), and InaD-like (Drosophila) (*INADL*). Top upstream regulators included heterogeneous nuclear ribonucleoprotein A2/B1 (HNRNPA2B1), class II trans-activator (CIITA), which regulates expression of MHC class II genes, the TP53 regulator structural maintenance of chromosomes protein 3 (SMC3), the cohesion component Rad21, and choline. In the predicted top regulator effects network the effector molecules were IFNG, IL1B, and TNF, that targeted a number of DEGs from the brain invasive *vs*. non-invasive comparison dataset (Fig 6). Downstream network functions were entitled, cell movement of myeloid cells, adhesion of blood cells, engulfment of cells, response of phagocytes, response of myeloid cells, binding of professional phagocytic cells, and recruitment of cells.

## Discussion

By utilizing whole transcript oligonucleotide arrays, we identified *DCC* as a candidate gene for tumor progression in grade I and II meningiomas using an initial multiple group comparison that was performed on each meningioma against the BN samples which served as control set. Subsequent grouping of the meningiomas according to their *DCC* expression levels resulted in a set of 416 DEGs between the *DCC* low expression and *DCC* high expression groups and the former one included the more aggressive tumors with the exception of one brain invasive meningioma that was categorized as a *DCC* medium expression tumor. DCC has not been so far identified in other microarray expression studies as a gene specifically associated with meningioma progression. In our opinion, this is based on the fact that *DCC* expression is apparently not a suitable discriminator between clinical benign and more aggressive meningiomas as *DCC* low expression was already observed in our study in those clinical benign meningiomas resembling with their expression profiles more aggressive tumors. As meningiomas are slow growing tumors, cell culture experiments on clinical meningioma samples with WHO grade I but *DCC* low expression profile could probably rule out the capacity of these tumors to progress into more aggressive cancer cells.

### Comparison with external expression data

A systematic review on 13 microarray studies on meningiomas reported that, due to the large number of markers present on the microarrays, a comparison between the studies to identify common gene signatures with implication in the tumor biology of meningioma is difficult [24]. Alternatively, this observed discrepancy between the results can be attributed to the fact that molecular and histopathological classification of meningiomas diverge and a number of microarray expression studies reported that unsupervised cluster analysis of meningiomas grouped the samples not according to histopathological classification schemes [25, 26]. A meta-analysis on seven microarray expression studies, representing 10 to 68 samples each, identified a set of 49 genes associated with progression and/or recurrence [3, 20, 27]. The majority of these genes were only shared between any two studies incorporated in the meta-analysis. In this regard, it is notable that from this 49er gene set, 14 genes (28.5%), namely *CKS2*, *CYR61*, *FGF7*, *FHL1*, *FOLR1*, *KIF23*, *LTBP2*, *MYLK*, *MYO5B*, *PHLDA1*, *PPAP2B*, *SERPINF1*, *SFRP4*, and *SVEP1* were shared with our set of 416 DEGs. In contrast, comparison of the 49er gene set with the 249 DEGs from the comparison between grade I and grade II meningiomas of our study (compilable from GSE77259 data set) resulted only in four shared genes.

### Lower expressed in *DCC* low expression meningiomas

*PDE1C* encodes a calmodulin dependent phosphodiesterase. This gene has a glioblastoma-derived neural stem cell signature that is significantly associated with poor prognosis of glioblastoma patients [28]. Glioblastoma multiforme (GBM) cell culture experiments demonstrated that PDE1C is a promoting factor for cell proliferation, migration and invasion which were inhibited by *PDE1C* silencing; however, a metadata analysis revealed that only 5.3% (32/596 cases) of primary GBM overexpress *PDE1C* [29]. *OLFM2* encodes a secreted glycoprotein of the member of the olfactomedin domain-containing proteins and is known to interact with NgR1 that modulates the functions of the NgR1 complex in axonal growth [30]. The function of *OLFM2* in cancer is not thoroughly elucidated. Among other genes, *GSTM5* had been identified with a hypermethylated promoter in GBM and accordingly was downregulated compared to control brain tissue [31]. In a combined cytogenetic and expression study, *GSTM5* was among those genes that were downregulated in meningiomas with complex karyotypes [32]. *PID1* was originally identified as an obese associated gene that, when overexpressed in 3T3-L1 adipocytes, impaired insulin-stimulated glucose uptake and phosphorylation of Akt and Irs1 [33]. Furthermore, cell culture experiments in medulloblastomas and gliomas revealed that cisplatin, etoposide, and vincristine induced transcriptional upregulation of *PID1* [34]. Of notice, higher *PID1* expression was positive associated with favorable prognosis of glioma patients. *SEMA6D* encodes a member of the semaphorin containing proteins and is expressed in different splice variants in a number of tissues including embryonic and adult brain where it is involved in axon guidance [35]. A metadata analysis on invasive breast carcinomas found a positive association between higher *SEMA6D* expression and favorable prognosis [36]. *INMT* had been identified as the top hub gene among 8000 genes which were analyzed by the TCNG database consortium on a set of 85 meningiomas [3, 37]. In prostate and lung cancer, *INMT* expression has been found to be downregulated [38].

### Higher expressed in *DCC* low expression meningiomas

*C5orf63* encodes a glutaredoxin-like protein that is mainly localized to mitochondria. Predicted interacting proteins include DENN/MADD domain containing 5B (*DENND5B*) and vesicular, overexpressed in cancer, prosurvival protein 1 (*VOPP1*) [39]. Its molecular functions in cancer are virtually unknown. *HIPK2* encodes a nuclear serine/threonine kinase that is a positive regulator of TP53 with a relation to tumor growth suppression and induction of apoptosis [40]. *HIPK2* amplification had been detected in pilocytic astrocytomas and HIPK2 overexpression in U87 human glioma cells resulted in enhanced cell growth [41]. *BHLHE40*, also known as DEC1, encodes a hypoxia inducible protein that is involved in diverse developmental and differentiation processes. A microarray expression analysis in mesenchymal gliomas found that expression of *BHLHE40* and a number of other transcriptional regulators of mesenchymal transition including *CEBPB*, *CEBPD*, and *STAT3* correlates with the extent of necrosis [42]. In breast cancer, expression of BHLHE40 correlated with invasiveness and its gene silencing in breast cancer cell lines resulted in reduced invasiveness [43].

### Higher expressed in meningiomas from females

*RTKN2* encodes a Rho-GTPase effector. Induced expression of *RTKN2* in HEK cells resulted in NF-KappaB dependent resistance to intrinsic apoptotic signals and *RTKN2* gene silencing in CD4+ lymphocytes lead to decreased BCL-2 expression [44]. Neuritin 1 is a neurotrophic factor involved in regulating synaptic plasticity and neuronal migration and has been identified as a downregulated gene in low grade meningiomas compared to normal meninges [45]. In contrast, a molecular genetic study in astrocytomas demonstrated that overexpression of neuritin 1 correlates with proliferation, apoptosis, and angiogenesis [46]. *SNORD114-26* is expressed from the 14q(II) snoRNA cluster that is highly expressed in brain and uterus. Elevated *SNORD114-26* expression was identified in a microarray study in dental follicle tissue in comparison to the periodontal ligament [47]. Of notice, besides *SNORD114-26*, the two small nucleolar RNAs *SNORD113-4* and *SNORD114-3* were also significantly higher expressed in meningiomas from females. Based on sequence similarities, LURAP1L is predicted to function as an adaptor implicated in regulating cell motility [48]. In osteoblastic cell line models, impairment of the estrogen receptor signalling pathway resulted in downregulation of *LURAP1L* [49]. In prostate cancer cell line PC-3, gene silencing of Toll-like receptor-9 (*TLR9*), reversed migration and invasiveness and resulted in downregulation of a number of genes including *LURAP1L* [50].

### Lower expressed in meningiomas from females

If transcription of the Y chromosome specific genes represents only a sex biased expression remains to be elucidated as for instance, for *DDX3Y*, *EIF1AY*, *KDM5D*, *NLGN4Y*, *RPS4Y1*, *USP9Y*, *UTY*, and *ZFY* a sex-biased gene level expression in one or more CNS regions has been reported [51]. *EPHA3* encodes a protein tyrosine kinase and was found to be highly expressed in the comparable aggressive mesenchymal subtype of GBMs [52]. Furthermore, *in vitro* experiments indicated that the receptor has the capacity to maintain tumor cells in a dedifferentiated state and its tumorigenic capacity was further demonstrated in orthotopic xenograft experiments. *PARVALB* and *CALB2* are critical modulators of intracellular calcium dynamics in neurons. In a microarray expression study on dorsal hippocampi of young female mice, administration of 17β-estradiol resulted in a number of DEGs including *Calb2* that was downregulated upon 17β-estradiol treatment [53]. SLC38A3 is an electroneutral, bidirectional glutamine transporter and is enriched in perisynaptic astroglial cell membranes. Strong immunoreactivity was revealed for SLC38A3 in high grade gliomas compared to metastases to the brain and control brain tissue [54].

### Higher expressed in invasive meningiomas

*CHL1* encodes a neural cell adhesion factor and has been identified in a microarray expression analysis as a considerably upregulated gene in schwannomas compared to control nerve cell samples [55]. Interestingly, besides its putative tumor suppressor functions in early tumor development, repression of *CHL1* has been shown in a cancer profiling array study to be associated with local tumor invasion in ovarian, colon, and breast cancer [56]. *RBFOX3* encodes a neuron-specific RNA binding protein that is functionally involved in miRNA biogenesis and neuronal developmental differentiation [57, 58]. PEX5L is expressed in the brain and its underexpression has been found in vestibular schwannomas associated with hearing loss [59]. Rab3D is a secretory small GTPase and *in vitro* and *in vivo* experiments established its critical function in cancer metastatic processes [60].

### Lower expressed in invasive meningiomas

The glucose transporter member *SLC2A12*, alias GLUT12, has been recognized as a major glucose transporter in cancer and therefore considered as a possible therapeutic cancer target [61]. *ABI3BP* was among other genes found to be upregulated in serum-differentiated GBM cells upon *SOX2* knockdown [62]. In thyroid cancer cells, reexpression of ABI3BP lead to reversion of the tumorigenic capacity of cells [63]. *SELPLG* encodes a selectin ligand and has essential functions in leukocyte trafficking during inflammation. Decreased *SELPLG* expression has been identified in recurrent compared to primary gliomas [64]. *INADL*, also known as PATJ, is a member of the cell polarity genes which have been recognized as critical factors in cancer progression [65].

In summary, this microarray expression study identified *DCC* as a candidate gene for early meningioma progression which could provide an explanation for the observed discrepancies between histopathological and molecular classification of meningiomas.

## Supporting Information

S1 Fig Semiquantitative RT-PCR on the five *DCC* high expression meningiomas, Jed13_MN, Jed26_MN, Jed40_MN, Jed43_MN, and Jed70_MN and the six *DCC* low expression meningiomas, Jed18_MN, Jed34_MN, Jed36_MN, Jed64_MN, Jed49_MN, and Jed58_MN. *DCC* primers located in exons 28 and 29 generated a PCR product of 187 bp and *B2M* primers generated a PCR product of 156 bp. Relative band densities of *DCC* compared to *B2M* varied in the *DCC* high expression group between 0.77 and 0.93 and in the *DCC* low expression group between < 0.01 and 0.09.
(TIF)

S2 Fig GO enrichment analysis for the 416 DEGs from the comparison *DCC* low *vs*. *DCC* high expression meningiomas. A, most prevalent in the cellular component domain were the categories extracellular matrix and extracellular region. B, in the molecular function domain, the most predominant categories were chemoattractant activity and molecular transducer activity. C, in the biological process domain, the most significant categories were biological adhesion and developmental process. The functional categories were scored by their *p*-values.
(TIF)

S3 Fig Merged network of the top five upstream regulators beta-estradiol, TGFB1, Tgf beta complex, LY294002, and dexamethasone that target a number of DEGs from the *DCC* low *vs*. *DCC* high expression comparison. Upregulated genes include, *ACKR3*, *ACTG2*, *ADAMTS5*, *APOD*, *BAMBI*, *BHLHE40*, *BMP5*, *BMP6*, *CKS2*, *COCH*, *DMTN*, *DSG2*, *FANCE*, *FGFR2*, *FOSL2*, *GDF15*, *GPRC5A*, *GRB10*, *HEY1*, *HIST2H2BE*, *ITGA2*, *KCNK2*, *LOC102724428/SIK1*, *MUC4*, *NR4A3*, *PCDH9*, *PHLDA1*, *PMEPA1*, *PODXL*, *RPRM*, *SSTR2*, *TMEM2*, *TP53I11*, *VEGFA*, and *ZNF385D*. Downregulated genes include, *ADAM12*, *ANGPT1*, *AQP1*, *AR*, *ARID5B*, *BCL2*, *CA12*, *CACNA1D*, *COL1A1*, *COL1A2*, *COL8A1*, *CTGF*, *CYP1B1*, *CYR61*, *DDIT4*, *ELN*, *ERBB4*, *FBLN1*, *FGF7*, *FHL1*, *FLRT2*, *FST*, *GAB1*, *GPR65*, *GSTM3*, *HLA-DQB1*, *HLA-DRB1*, *HOXC5*, *HOXC6*, *IL33*, *ITGA11*, *ITGA4*, *LIPC*, *LOX*, *LTBP1*, *LTBP2*, *MATN2*, *MFGE8*, *MME*, *MMP2*, *MSR1*, *MT1M*, *MXRA5*, *MYLK*, *NAV3*, *NCAM1*, *NNMT*, *NOV*, *OLFM2*, *PAPPA*, *PIK3R1*, *PLCB1*, *PLPP3*, *PLSCR4*, *PROM1*, *PTGER2*, *PTPN20*, *PXDN*, *PYGL*, *RASSF2*, *RBP4*, *ROR1*, *SCG2*, *SEMA3F*, *SERPINE2*, *SERPINF1*, *SLC23A2*, *ST8SIA1*, *SVEP1*, *TGFB3*, *TGFBI*, *TGM2*, *THBS1*, *TLR2*, *TPD52L1*, *TRIM2*, *TSPAN7*, and *VCAM1*.
(TIF)

S1 Table Genes differentially expressed between *DCC* low and *DCC* high expression meningiomas. (XLSX)

S2 Table Genes differentially expressed between meningiomas from female and male patients. (XLSX)

S3 Table Genes differentially expressed between brain invasive and non-invasive meningiomas. (XLSX)

## Abbreviations

ANOVA: analysis of variance
BN: brain normal
DCC: deleted in colorectal cancer
DEG: differentially expressed gene
GBM: glioblastoma multiforme
GO: gene ontology
PCA: principal component analysis
