# Supplementary figures and images for: Microarray Expression Data Identify DCC as a Candidate Gene for Early Meningioma Progression
Source: PLoS One. 2016 Apr 20;11(4):e0153681. doi: 10.1371/journal.pone.0153681 (PMC4838307; doi:10.1371/journal.pone.0153681)

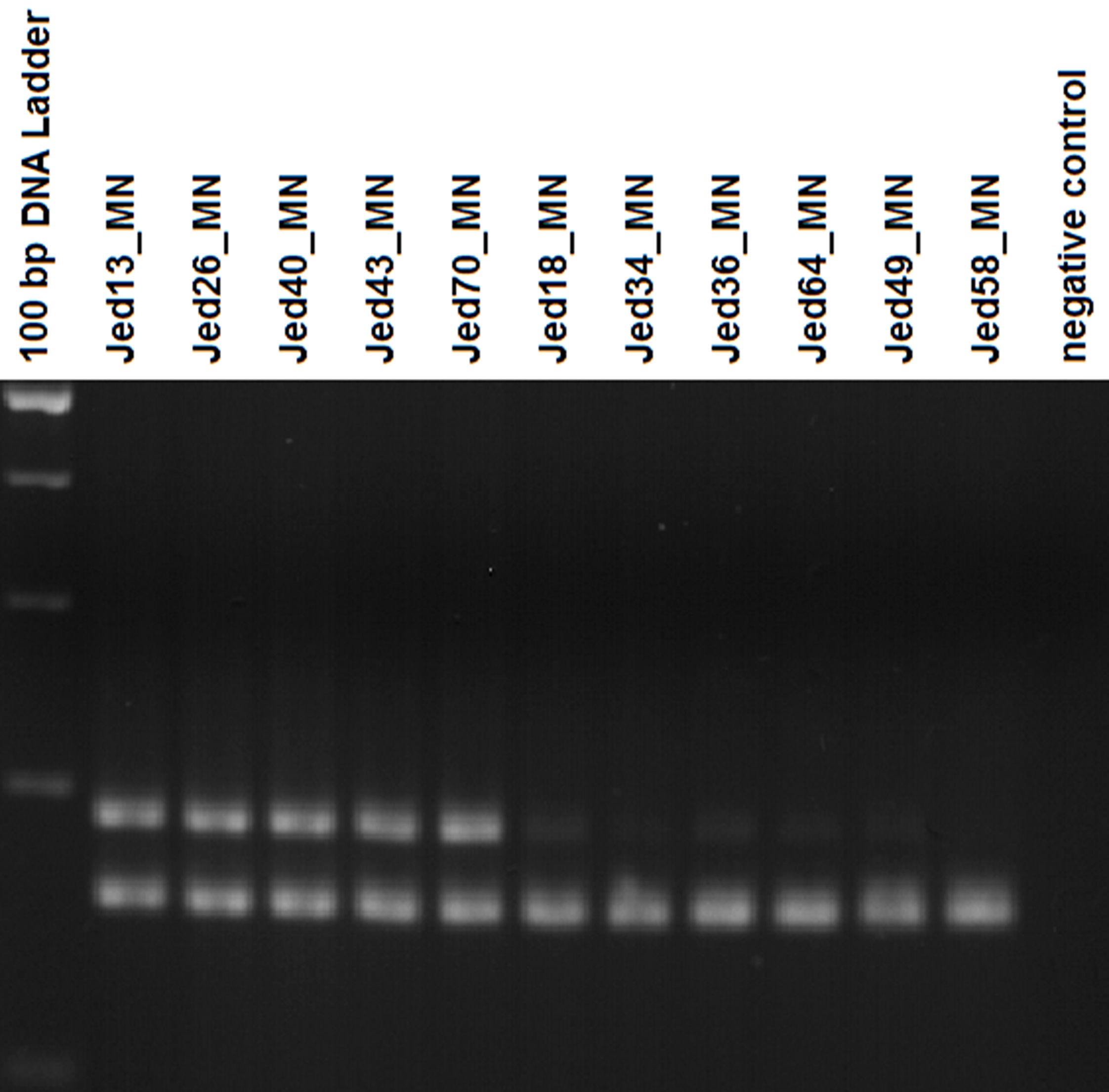

Supplement: S1 Fig — DCC primers located in exons 28 and 29 generated a PCR product of 187 bp and B2M primers generated a PCR product of 156 bp. Relative band densities of DCC compared to B2M varied in the DCC high expression group between 0.77 and 0.93 and in the DCC low expression group between < 0.01 and 0.09. (TIF) [file pone.0153681.s001.tif]

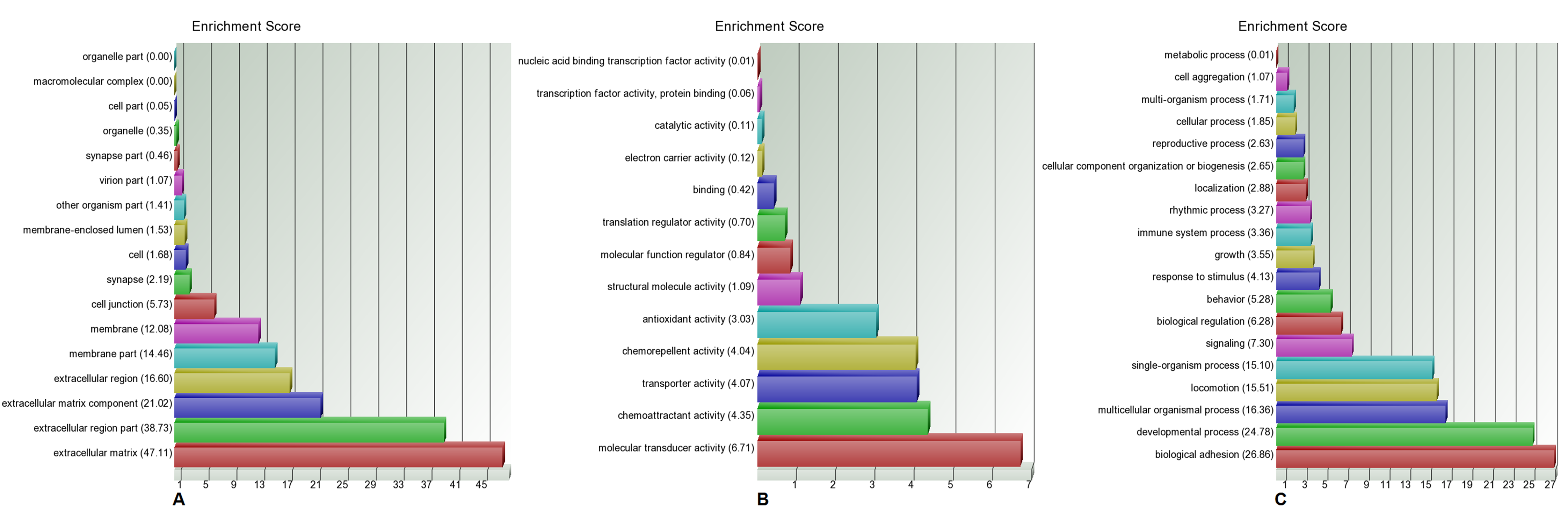

Supplement: S2 Fig — A, most prevalent in the cellular component domain were the categories extracellular matrix and extracellular region. B, in the molecular function domain, the most predominant categories were chemoattractant activity and molecular transducer activity. C, in the biological process domain, the most significant categories were biological adhesion and developmental process. The functional categories were scored by their p-values. (TIF) [file pone.0153681.s002.tif]

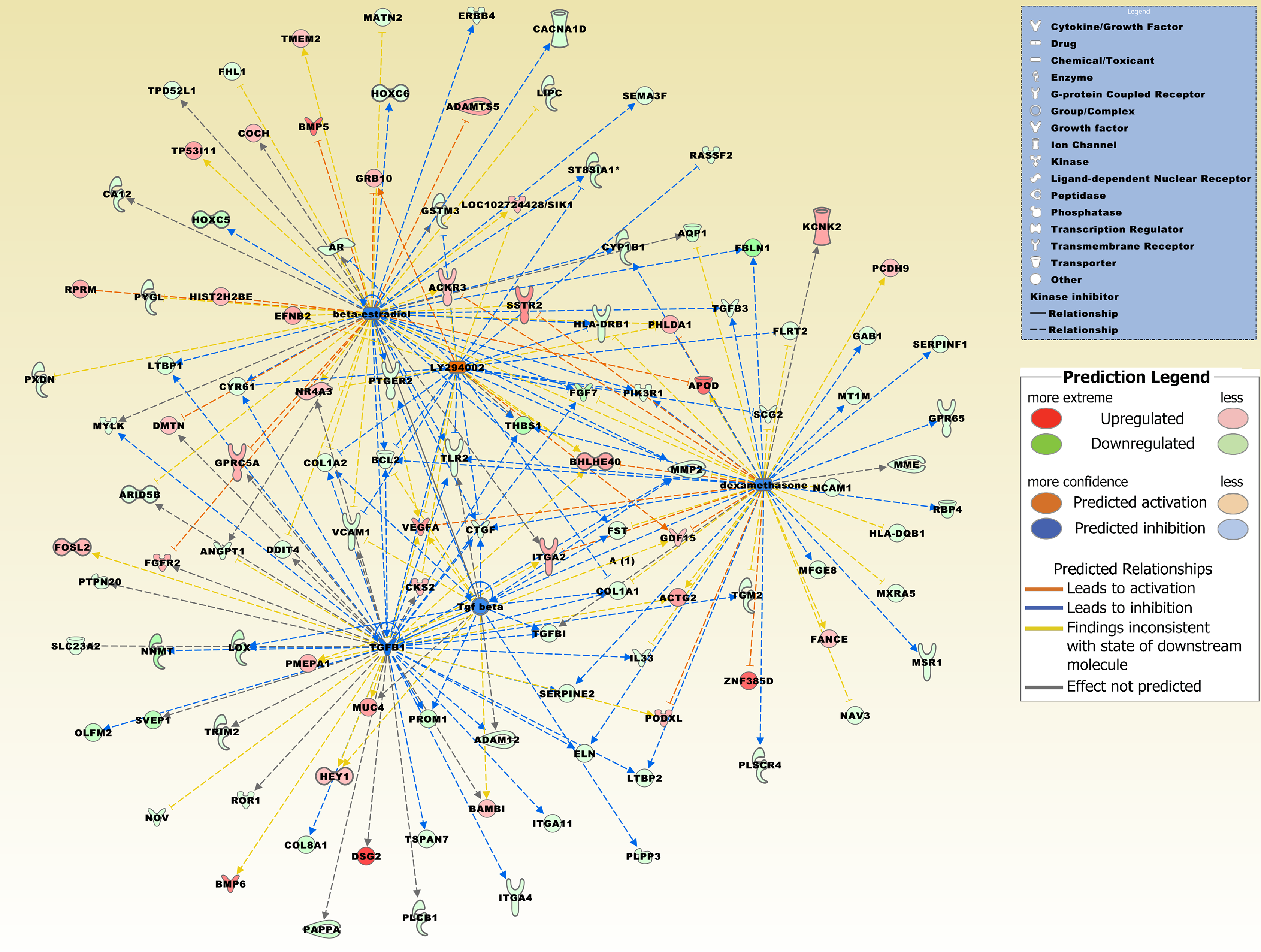

Supplement: S3 Fig — Upregulated genes include, ACKR3, ACTG2, ADAMTS5, APOD, BAMBI, BHLHE40, BMP5, BMP6, CKS2, COCH, DMTN, DSG2, FANCE, FGFR2, FOSL2, GDF15, GPRC5A, GRB10, HEY1, HIST2H2BE, ITGA2, KCNK2, LOC102724428/SIK1, MUC4, NR4A3, PCDH9, PHLDA1, PMEPA1, PODXL, RPRM, SSTR2, TMEM2, TP53I11, VEGFA, and ZNF385D. Downregulated genes include, ADAM12, ANGPT1, AQP1, AR, ARID5B, BCL2, CA12, CACNA1D, COL1A1, COL1A2, COL8A1, CTGF, CYP1B1, CYR61, DDIT4, ELN, ERBB4, FBLN1, FGF7, FHL1, FLRT2, FST, GAB1, GPR65, GSTM3, HLA-DQB1, HLA-DRB1, HOXC5, HOXC6, IL33, ITGA11, ITGA4, LIPC, LOX, LTBP1, LTBP2, MATN2, MFGE8, MME, MMP2, MSR1, MT1M, MXRA5, MYLK, NAV3, NCAM1, NNMT, NOV, OLFM2, PAPPA, PIK3R1, PLCB1, PLPP3, PLSCR4, PROM1, PTGER2, PTPN20, PXDN, PYGL, RASSF2, RBP4, ROR1, SCG2, SEMA3F, SERPINE2, SERPINF1, SLC23A2, ST8SIA1, SVEP1, TGFB3, TGFBI, TGM2, THBS1, TLR2, TPD52L1, TRIM2, TSPAN7, and VCAM1. (TIF) [file pone.0153681.s003.tif]
